# “The general public will never truly know what it feels like”: Exploring the Lived Experiences of Persons Affected by Leprosy in Sindh, Pakistan – A qualitative study

**DOI:** 10.1371/journal.pmen.0000453

**Published:** 2025-11-05

**Authors:** Sophie C.W. Unterkircher, Abdul Salam, Isabel Fernandes, Anil Fastenau

**Affiliations:** 1 Department of Global Health, Institute of Public Health and Nursing Research, University of Bremen, Bibliothekstraße 1, Bremen, Germany; 2 Marie Adelaide Leprosy Center (MALC), Mariam Manzil, A.M. 21, Off Shahrah-e-Liaquat, Saddar, Karachi, Pakistan; 3 German Leprosy and Tuberculosis Relief Association (GLRA/DAHW), HQ, Raiffeisenstraße 3, Wuerzburg, Germany; Kathmandu Institute of Child Health, NEPAL

## Abstract

This study examines the impact of leprosy on those affected by leprosy in Sindh, Pakistan, exploring their care-seeking behavior, treatment experiences, well-being, stigma and coping mechanisms. By highlighting the lived experiences of persons affected by leprosy (PAL), this study aims to inform strategies that address not only the biological aspects of leprosy, but also the social and psychological challenges faced by this marginalized population. Fifteen in-depth interviews were conducted with persons affected by leprosy from Sindh, Pakistan. A combined inductive and deductive thematic analysis approach was applied inspired by the “conceptual framework of the bio-psycho-social impact and coping mechanisms of patients” by Han et al. Participants reported delays in diagnosis of up to twenty years, significantly influenced by limited awareness and socio-economic barriers. These delays increased the risk of physical disability and worsened mental health problems such as depression and anxiety, largely fueled by stigma and misinformation. While both genders experienced stigma, women were more likely to express their mental health problems, highlighting gender differences in discussing such issues. Peer support and community storytelling emerged as effective strategies to mitigate stigma, while the role of religious beliefs shaped both societal perceptions and personal coping mechanisms. The findings highlight the need for comprehensive, culturally sensitive interventions that address the bio-psycho-social challenges faced by PAL. Implementing people-centered strategies that use storytelling, community engagement and digital health tools are essential to reduce delays in diagnosis and improve the overall well-being of PAL. Further research is needed to refine these interventions and ensure that they are aligned with the needs and beliefs of those affected by leprosy.

## Background

First reported approximately 4000 years ago, the chronic infectious disease leprosy remains endemic in more than 120 countries to this day [1, 2]. Although, the decline in new leprosy cases has been gradual, with 112 countries reporting less than 1000 new cases annually, countries like Brazil, Indonesia and India continue to report more than 10 000 new cases yearly [2]. Leprosy, also known as Hansen’s disease, is caused by Mycobacterium leprae and nowadays classified as a Neglected Tropical Disease (NTD) [2]. Infection may result in physical symptoms affecting the skin, peripheral nerves, mucosa, upper respiratory tract and eyes [3, 4]. Despite the fact that leprosy is curable and multi-drug therapy (MDT) is provided free of cost, persons affected by leprosy (PAL) may experience permanent disability. That is due to delayed diagnosis, often caused by limited access to care, financial constraints, low awareness and stigma [3, 5].

Stigma has stuck to the disease for centuries, enforcing prejudice, segregation, suppression of those affected and fear in society [6]. PAL have long been described as unclean, cursed, poor, and overly contagious, with current sayings still referring to such unwarranted claims, like “Don’t treat me as if I have leprosy” [6]. In turn, the mental health and quality of life of PAL may further be affected by facing social stigma. Besides, studies have found that stigma and mental health issues can be deterrents to care-seeking and treatment adherence, especially in low-endemic settings [5, 7]. While a number of high-endemic countries have developed national leprosy elimination programs, low-endemic settings like Pakistan redirected their national priorities toward other pressing health concerns [8–10]. Despite this, the lower-middle income country Pakistan, with around 58,500 registered cases and 200–300 new cases annually (as of 2020), has made tremendous progress toward leprosy elimination [11]. In the absence of a national leprosy control program, two non-governmental organizations (NGOs) Marie Adelaide Leprosy Center (MALC) and Aid to Leprosy (ALP) are primarily responsible for leprosy control efforts and care provision in the country [12,13]. A specific focus is placed on the province of Sindh, which accounts for 38 percent of Pakistan’s leprosy cases, making it a key region to be investigated in the context of current challenges faced by PAL [14]. In order to develop an understanding of what it is like to live with leprosy in a low-endemic setting like Pakistan, where the disease seems to be forgotten [12], this study gives voice to those affected by leprosy. By exploring their care and treatment journey, well-being, stigma experiences and coping mechanisms, this study is the first to delve into the perspectives of PAL in Pakistan since 1989 [15]. Inspired by the “conceptual framework of the bio-psycho-social impact and coping mechanisms of patients” developed by Han et al. [16] and the two dimensions of stigma; enacted stigma (experiences of discrimination) and internalized stigma (adopting negative self-perceptions) [17], lived experiences were explored comprehensively. While this topic may be seen as longstanding, we cannot shift our focus away from it, until all people have realized their right to health. It is essential that we do not overlook the issue of equity, particularly with regard to neglected diseases and those further neglected by it. In line with the WHO Global Leprosy Strategy 2021–2023 “Towards Zero Leprosy” and the Sustainable Development Goals (SDGs), the insights from those affected by leprosy are invaluable to informing Pakistan’s efforts to eliminate leprosy and empower PAL [18,19].

## Methods

### Study design

**Fig 1:**
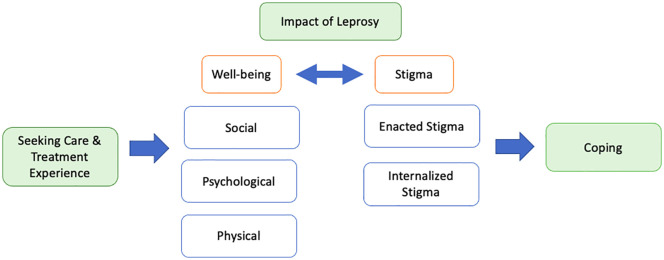
Conceptual Framework on the wellbeing, stigma and coping mechanisms of PAL (created by author).

We conducted a purposive, cross-sectional, qualitative study exploring the lived experiences of PAL. The study design was guided by two dimensions of stigma, including 1) enacted stigma (“experiences of discrimination, stereotyping, and/ or prejudice from others in the past or present due to a stigmatizing condition or behavior”) and 2) internalized stigma (“endorsing negative feelings and beliefs associated with the stigmatized condition and applying them to the self”) [17]. Along with that, the “conceptual framework of the bio-psycho-social impact and coping mechanisms of patients” developed by Han et al. was adapted to contextualize the broader effects of leprosy on PAL’s lives (see Fig 1) [16]. This theoretical grounding helped maintain focus during interviews while allowing participants to express experiences in their own ways. During the interviews, participants were encouraged to describe their emotional and social experiences in their own words. Terms such as “depression” or “anxiety” reflect the participants’ self-descriptions and do not constitute clinical diagnoses. Similarly, we did not assess or classify the severity or stage of leprosy, due to the study’s focus on subjective lived experiences rather than medical assessment.

### Study setting

The study focused on the region of Karachi, Sindh. The southeastern region is the second largest province of Pakistan by population and reports the majority of leprosy cases throughout the country [20]. Interviews were conducted in a private consultation room within the in-patient facility of the NGO MALC, headquartering in Karachi.

### Participants

The recruitment and interviewing of participants took place between October 9^th^ until October 18^th^, 2024. In collaboration with the medical personnel at MALC, a purposive sampling method was employed to recruit participants who could provide insights into lived experiences of leprosy [21]. Inclusion criteria included 1) Persons directly affected by leprosy 2) being above eighteen years of age 3) currently admitted to MALC’s in-patient facility 4) willingness to participate, and 4) ability to share experiences and communicate in an interview setting. Furthermore, authors aimed to interview an equal number of men and women affected by leprosy.

### Data collection

Fifteen semi-structured interviews were conducted by the author together with an Urdu-speaking translator. To ensure the interviewees’ comfort and privacy, in-depth-individual interviews (IDIs) were administered in a separate consultation room familiar to them. To respect cultural norms regarding the social roles of men and women, interviews with women were conducted together with a female translator. For men, it varied between a male and female translator. Overall, interviews averaged a duration of thirty minutes.

In collaboration with the co-authors, an interview guide was developed, informed by the two dimensions of stigma, Han et al.’s conceptual framework and established quantitative leprosy scales (S1 Table 2). Questions were contextualized, by excluding those not culturally appropriate, neutrally rephrasing leading questions, and adding new questions to comprehensively capture participants experiences. The initial two interviews also posed as pilot interviews, allowing the author to refine interview questions for clarity and relevance.

### Data analysis

Interviews were audio recorded and transcribed using the external artificial intelligence (AI) software noScribe. Transcripts were pseudonymized by the author and data was analyzed using a thematic analysis approach by Braun & Clarke [22]. Three iterative rounds of inductive and deductive coding were completed, supported by the qualitative analysis software MAXQDA 2022. Initial codes were drawn from the applied frameworks [16, 17] to capture anticipated themes, while openness to new, unexpected themes was maintained. This balance allowed the framework to structure analysis around overarching topics without constraining the emergence of novel insights.

### Ethical approval

Ethical approval was obtained from both the Ethical Review Committee of MALC (MAC/ERC/2024/01) in February 2024 and of the University of Bremen (2024-10) in April 2024. The conducted research adheres to PLOS Inclusivity in Research standards (S1 Checklist). All data is pseudonymized and stored on a secure, password protected device and will be deleted after five years. Participants were provided with an information sheet translated into Urdu outlining the purpose of the study. Informed consent was obtained in written form using a consent document translated into Urdu. Participants indicated their consent by either signing their name or providing a thumbprint.

## Results

### Characteristics of participants

A total of fifteen PAL participated in the interviews, divided into seven male and eight female PAL. Interviewees ranged in age from eighteen to over seventy years, with the majority between fifty and seventy. Participants had been living with leprosy for an average of twenty-five years. The majority were married and left their job because of leprosy or old age (Table 1).

**Table 1:** Characteristics of study population.

Persons Affected by Leprosy	Age	Sex	Years since leprosy diagnosis	Marital status	Occupation
PAL1	60-70	Female	40 years	Married	Housewife
PAL2	58	Male	19 years	Married	Mechanic*, Sales
PAL3	62	Female	30 years	Widow	Sewer
PAL4	60	Male	24 years	Married	Secretary*
PAL5	70+	Female	30 years	Widow	Sewer*
PAL6	40	Female	30 years	Married	Cleaning lady*
PAL7	70	Male	34 years	Married	Sewer, Sales*
PAL8	75	Male	25 years	Married	Gate keeper*
PAL9	50-60	Female	18 years	Married	Teacher *
PAL10	78	Female	25 years	Widow	Factory*
PAL11	78	Male	22 years	Married	Sales*
PAL12	45	Male	3 years	Married	Sales*
PAL13	50	Male	40 years	Widow	Unknown
PAL14	18	Female	2 years	Unmarried	Unknown
PAL15	Unknown (ca.70+)	Female	Unsure (a long time ago)	Widow	Factory, Sales*

*Left job because of leprosy or old age

Results were structured into five main themes with respective sub-categories: 1) Seeking care and treatment experiences 2) Facing the impact of leprosy on physical, mental and social well-being 3) Experiencing stigma 4) Coping with leprosy 5) Direct messages from PAL to peers and society (Fig 1).

### Seeking care and treatment experiences

As displayed in Table 1., participant’s history of diagnosis averaged 20–30 years. Many interviewees were not able to identify the exact origin of their leprosy infection due to experiencing delays in diagnosis and their prolonged history with the disease. Two suspected it to have originated from a rat bite due to sleeping on the floor or an infected wound contracted from the ocean. The majority of PAL expressed that they “eventually came in” for the right treatment, after wounds failed to heal, conventional over-the-counter-antibiotics proved ineffective, misdiagnoses were encountered, and enough funds were saved to afford care.

*“A lot of people just do not have the luxury to visit a doctor, and me myself. Like three years ago when I got it, I just basically didn’t have the resources to go and see a doctor, so I thought I might as well just start doing something at home about it and take care of it as soon as possible.” (PAL 12, male)*

*“I went to multiple doctors [with them] saying, “you have an allergy”, then I went to a*

*dermatologist later and only then learned about having leprosy. ” (PAL 6, female)*

Furthermore, many PAL explained that leprosy is not commonly discussed, especially in rural areas, which leads to a delay in care. As one participant stated:

*“A lot of people even if they are infected with the disease, if they do have it, they do not have enough awareness about it, so holding camps in very tribal and rural areas, like in Sindh for example you have the smaller cities, and you might need to create more awareness there.” (PAL 2, male).*

In some instances, friends and family, the priest, local doctors, community members or Ricksha drivers encouraged them to seek care. One woman narrated:

*“Once there was a rat that bit me and it left a spot on my leg and when a friend or community members saw it they said it doesn’t look normal and I should get it checked out and that’s when all of this happened.” (PAL 1, female)*

While others would immediately visit the right centers and skin camps after noticing wounds or be approached by healthcare workers (HCW) in their homes.

When asked about their experiences with receiving leprosy treatment, all PAL voiced their appreciation for the supportive nature, good care, comfort and their trust towards the medical personnel at MALC.

*”I have been to many places, and while they have been good enough, it was always from one place to another, with them saying “go here, go there”, and ever since coming here, haven’t really felt the need to go anywhere else.” (PAL 1, female)*

PAL explained that they are happy to be provided with medication, food, clothes and support for day-to-day tasks, with some even stating that they “ *[…]* *don’t trust anybody except MALC doctors, I will always come to MALC hospital now even for a flu, or cough.*” *(PAL 5, female).*

Another man explained:

*“*[…] but I feel over here it is a little more like it will help cure the disease rather than just living out there and going through the motions.*”* *(PAL 12, male)*

### Facing the impact of leprosy on physical, mental and social well-being

#### Noticing Leprosy/ Effects on Body.

Various insights were shared, expressing how PAL first recognized that something was “different” in their body. Common symptoms were described to be burning skin, random sores, round patches, swelling, vomiting, losing hair, fevers, loss of sensation and numbness in hands and feet as well as pigmentation, where the “*face turned black*” *(PAL 10, female).* Hence, some have started to encounter difficulty when trying to walk, experience exhaustion quickly and require glasses due to vision impairment.

*“First I realized that I felt weakness all over my body and then it started that I can’t feel the ulcers and I can’t walk properly because of the numbness in my feet.” (PAL 7, male)*

One young woman explained that she used to go on shopping trips with her father, and every now and then her shoes would slip of her feet, due to swelling, although she was not in pain, she could not explain what was happening to her *(PAL 14, female).* Another man shared that he used to play sports as a good athlete and “*then started to get these leg pains and all of that*” *(PAL 4, male)*, which were later mistaken as sport injuries.

#### Impact on mental health.

Next to several physical impacts, often intensified by delay of diagnosis, PAL outlined the effects of leprosy on their mental health. *“Why me, why did it happen to me?”* was asked many times, with PAL describing them to feel “*completely lifeless” (PAL 15, female)* at times, depressed, mentally exhausted, and *“furious” (PAL 5, female).*

*“Leprosy has mentally exhausted me so much, that I wish leprosy on no one. “(PAL 6, female)*

Interviewed men described themselves to have been under immense stress due to worrying about how to take care of their family, stating very emotionally that “*the stress was more about what’s my family going to do? How is my family going to react and how are they going to manage?” (PAL 12, male)*. Some PAL expressed great sadness due to losing their jobs, encountering hate, fear and complaints from their family and friends. However, in a few instances, PAL experienced no anxiety or depression and, in their words, they “*simply accepted it*”, while others did not want to talk about it.

#### Impact on the day-to-day (social).

Given the physical and psychological effects of leprosy on PAL, numerous implications are faced socially on a day-to-day basis. Interviewees commonly expressed the hardship they faced regarding finding, keeping, or losing job opportunities, due to disability, numbness, early exhaustion or being discriminated against.

*“Everyone sees my ulcer, so they ignore me, […] wherever I go for a job, they always say you are disabled, you won’t be able to do the job properly.” (PAL 8, male)*

Hence, families are confronted with economic burden, as one man outlined in tears:

*“My son hasn’t been going to school in the last month because they haven’t been able to afford it with the disease.” (PAL 12, male)*

In order to find employment, regardless of being affected by leprosy, many PAL explained to have shifted professions. For instance, going from sewing clothes to selling products or standing on their feet for fifteen hours, bearing the pain to make a living. Another woman revealed that she has not been able to facilitate marriage for her sons, as she is not able to raise enough money because of her illness. Along with that, daily tasks, such as cooking meals, sweeping the house, moving around and praying present as a challenge.

*“[…] I used to pray […] 5 times a day, every day for almost my whole life. And now I do it as well, but only at night, because it is more difficult.” (PAL 1, female)*

*“With leprosy I find it hard to do my day-to-day tasks, at my house I can’t cook meals, my brother needs to bring food from the hotel he works at. Even when I sweep the house, it gets very difficult for me to move around on a daily basis. I had problems with my vision, so they had to give me glasses. So, it is all those things, all those adjustments I had to make because of the pain I go through due to leprosy.” (PAL 14, female)*

### Experiencing stigma

#### Encountering reactions and discrimination (enacted stigma).

In terms of encountering reactions from social networks, some indicated to have never faced discrimination, where people would neither ask about their condition nor pay attention to it altogether. However, several interviewees shared counter examples, displaying various facets of leprosy-related stigma. It was described that after diagnosis and showing signs of leprosy, people used to fear their presence, laugh at them, attack them verbally and question their abilities to work or take care of their family. A man shared:

*“People were saying, you are disabled, how are you going to work, how are you going to take care of your family?” (PAL 7, male)*

Next to that, one woman described that it made her furious:

*“There were people who didn’t want to talk to me, sit with me, eat with me, showing disrespect and discrimination towards me.” (PAL 5, female)*

Especially in regard to marriage or dealing with in-laws, a lot of challenges were faced for both men and women, whereas only one man shared his experience about it. He narrated that when he first got married, he was met with a lot of hate from his wife, who would *“[…] complain a lot to her father, asking why did you get me married to this man, he is sick, he is this, that” (PAL 13, male)*, making him very emotional. Another woman expressed that after receiving her leprosy diagnosis, her mother-in-law would *“[…] start saying bad stuff”* and not *“[…] treat her nicely anymore” (PAL 3, female)*, making her feel very depressed. Further, one affected woman described that her daughter-in-law refuses to have her stay with her, worried she might pass it on to her, saying *“[…] Oh you will pass this illness on to us, don’t eat with us, go away, stay away from us”*, thus she has not seen her in a long time *“[…] she doesn’t even come to the door step.” (PAL 1, female)*

Generally, one man noted that he has received so much hate over the years and people still treat him differently although decades have passed.

*“They hate us with no reason.” (PAL 13, male)*

#### Acting on basis of stigma (internalized stigma).

Due to the above-described experiences of discrimination and stigma, negative feelings and sheltered behavior were commonly endorsed among PAL. Five participants explained that they stay at home or keep themselves isolated. Reasons included fears of spreading germs, feeling that they were not allowed to leave the house when they had ulcers, and wanting to avoid further discrimination. One woman stated that she asked God for forgiveness, indicating the leprosy infection to be her fault.

Due to stigma, some felt it was too “risky” to disclose their leprosy diagnosis, choosing to hide it for years out of fear of rejection, isolation and hatred.

*“I hide the fact that I have leprosy, even from my daughter in law. So, when she for example notices, I just say no, no it is fine.” (PAL 13, male)*

“*You should just hide it because you are not spreading it, because you will only get a negative response from others.” (PAL 7, male)*

In some instances, PAL would only tell close family members about their leprosy diagnosis.

*“I can’t talk around the people, people stay away from us, so only my family knows, but normally the neighbors don’t know about it.” (PAL 9, female)*

A handful of interviewees mentioned that they easily disclosed their diagnosis to family, friends and community members, others would share, that people knew about it only because doctors would come and check on them.

### Coping with leprosy

#### Support from family and peers.

To cope with leprosy, PAL shared several strategies that helped them to maintain hope and positivity on their journey with leprosy. A number of participants find comfort in families, friends and sometimes even in medical personnel. PAL explained that they also support one another. As one participant expressed *“[…] no person should be left alone, [we are] all going through this together”*, noting that he finds comfort in talking with others who have lived through the same experiences *(PAL 2, male).*

*“I get a lot more helpful advice now, rather than just getting things from people who haven’t lived with the experience, I get a lot of advice, and am able to give advice.”(PAL 12, male)*

Another PAL narrated:

*“I talk to other patients, get to know that they are also having their problems with it, so it’s not just me, which feels good. All the patients that used to be here for a long time or have worse complications would talk to new patients and counsel them, which is comforting for them.” (PAL 7, male)*

Seeing other PAL with worse conditions also seems to have helped participants realize that their condition “*is not so bad in comparison to others*” (*PAL 11, male).* Three other interviewees voiced a desire to connect with other PAL to talk about similar experiences. While another PAL, who prefers to mostly keep to himself, expressed that he tries to support other PAL, who are *“freaking out about their symptoms*”, reassuring them that *“[…] even if it is leprosy, there will be doctors who will give you treatment for it and help you get through it.” (PAL 13, male)*

#### Religion.

Next to social support, religious and spiritual beliefs play a central role in the way PAL cope with leprosy. The majority of participants expressed that they felt that leprosy was *“God’s plan”* or *“God’s will”* for them. One PAL explained *“[…] again, God would not have given it to you, if he would not have thought you could get through it.” (PAL 14, female)* Thus, the majority of PAL perform prayers every day, asking God to make things better, take away the disease and to help them get through it.

*“Whatever comes my way I, will be okay by just praying to God.” (PAL 1, female)*

Furthermore, gratitude towards God is expressed for pointing them in the direction of MALC, for having supportive children, experiencing pain relief and not having it as bad as other people. On the one hand, most PAL are hopeful to get better and make it their mission not to dwell on the past, but rather look towards the future. One man expressed:

*“I don’t really think about it at all, whatever comes to me, I deal with at that point and that’s it, because when I take a moment to stop and think about it then I will go into a spiral and I don’t really want to think about it. So, I just look towards the future.” (PAL 2, male)*

On the other hand, four participants stated that they do not believe they can be cured or get better, and that because of their old age, they will most likely die with it. 

### Direct messages from PAL to peers and society

**Fig 2:**
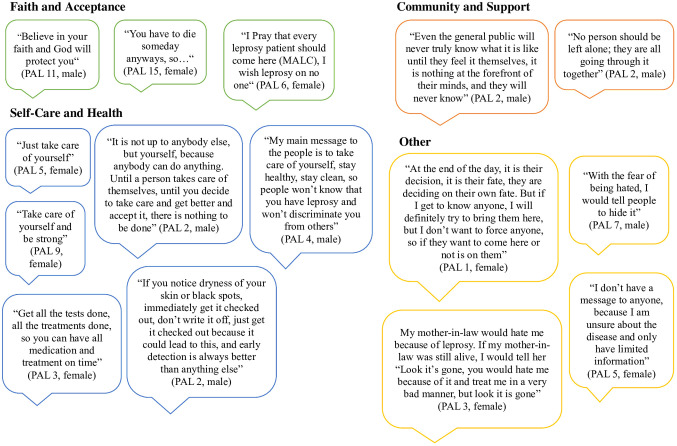
The Voices of PAL.

To empower the voices of persons affected by leprosy participating in this study, we gave participants the opportunity to share direct personal messages to peers and society during the interview. Messages refer to faith and acceptance, self-care and health, community and support and other miscellaneous topics (Fig 2).

## Discussion

This study has shown the impact of leprosy on the lives of those affected by it. The findings have allowed us to understand the interconnectedness between bio-psycho-social wellbeing, stigma and coping mechanisms through the eyes of PAL.

### Addressing psychosocial impacts of leprosy

Overall, PAL narrated that lack of awareness about leprosy, along with economic and geographic barriers affected their care-seeking behavior, leading to diagnostic delay by up to twenty years. This suggests a diagnostic delay almost twice as long as that reported in two studies conducted in more endemic areas of Indonesia [23,24]. This delay not only increases the risk of physical disability, but also exacerbates mental health problems and stigma [5]. This study further demonstrated that interviewees commonly reported symptoms of depression, anxiety and mental exhaustion. These symptoms were a response to the physical, social and psychological impact leprosy had on their lives.

PAL experienced social exclusion and discrimination, fueled by widespread misinformation and stigma. They also faced difficulties in finding employment or marriage partners, which contributed to negative feelings, low self-esteem and social isolation. According to a systematic review [25], PAL with leprosy-related anxiety or depression are less likely to seek care, adhere to treatment or participate in social reintegration programs. This underscores the importance of integrating psycho-social interventions into leprosy control efforts. However, it is important to bear in mind that, similar to leprosy, mental health struggles are often stigmatized, therefore PAL may hesitate to seek or accept help for their mental health [26,27].

In this study, it was observed that women were more likely, than men, to share insights about their hardships and mental health problems. This may reflect broader patterns, as women are generally at higher risk of mental health problems and face more discrimination and stigma in a patriarchal society like Pakistan [28, 29]. While the study did not focus on gendered trends, participants’ gender was noted throughout to contextualize individual narratives. It is important to recognize that while women need to be empowered within their social roles, it is equally important to overcome the stigma that discourages men from discussing their mental health [30]. Especially, Pakistan documents one of the highest mental illness rates in the world [28]. While mental health is often of secondary concern in disease-specific programs, it poses as a key factor for those affected by leprosy on their journey to recovery and reintegration into society [26,27]. Hence, it is crucial to spread more awareness not only on bio-medical aspects of leprosy, but also on its mental health complications.

Consequently, this study highlights that mental health and well-being should be an integral part of leprosy elimination efforts in Pakistan [26]. A study from Nigeria showed that integrated mental health and NTD care models demonstrated the feasibility and acceptance of including stigma-support into clinical services at the primary care level [31]. These interventions should be rigorously evaluated for their effectiveness in reducing stigma and improving mental health outcomes in the context of Pakistan [32]. Further research is needed to explore the interconnectedness between gender, stigma and mental health within the country.

### Empowerment, coping and social integration

Overall, it is crucial to involve the voices of those affected by leprosy into the decision-making processes and development of interventions and policies. As one PAL expressed: “Even the general public will never truly know what it is like until they feel it themselves, it is nothing at the forefront of their minds, and they will never know.” (PAL 2). HCW and community leaders can support PAL in sharing their stories through social media outlets, storytelling events at mosques or churches, educational institutions and local clinics.

Following that, a study conducted in Nigeria and the Democratic Republic of Congo employed a participatory method called “photovoice”. Participants were given cameras or smartphones to capture their daily lives or personal stories, to humanize their experience and control the narrative of how their stories were told. Photographs later formed community exhibitions, and advocacy efforts [33]. Participant-driven, community-based rehabilitation (CBR) interventions like these are not only cost-effective but also empowering to PAL. As highlighted by the interviewees, sharing their stories with other PAL helps reduce symptoms of anxiety, alleviates feelings of isolation, and provides hope.

Creating self-care groups by training PAL and involving them as counsellors, awareness-raisers and educators, has similarly proven effective. For example, a pilot study in India tested the Basic Psychological Support for NTDs (BPS-N) model, where PAL and persons affected by lymphatic filariasis were trained as peer supporters to deliver structured psychological support. This resulted in improved mental well-being, reduced depressive symptoms and enhanced social integration [34]. Studies from Indonesia support the use of such approaches, while pointing out that more research is required focusing on sustainability, establishing uniform boundaries and gender dynamics [35–38].

Moreover, this study displayed that religion can reinforce leprosy stigma by influencing societal perceptions and moral judgments, yet also offer individuals guidance and support for coping. Next to family and peer support, most of the participants described religion as their primary coping mechanism. It provided comfort by helping them look beyond their immediate circumstances. Although, misinformation caused by religious and spiritual beliefs need to be addressed by people working in the field of leprosy, it is important to respect and integrate the religious and spiritual beliefs of PAL into their treatment and recovery journey [25]. This may also strengthen the relationship between PAL and HCW, which was found to improve the self-management and treatment satisfaction across a number of studies from Nepal, Brazil, Ghana and Indonesia [25,35,39–41].

Furthermore, participants emphasized the need to focus outreach efforts on remote, tribal and overall rural areas. Reaching people in more remote areas can be assisted by digital health tools, such as portable and offline adaptable tele-dermatology services and diagnostic algorithms, streamlining a more cost-effective and easier approach to help with education, counseling, preliminary diagnosis and referral [42]. Although the usage of tele-medicine does not entirely replace an in-person care, it serves as a valuable complement, enhancing access to healthcare and timely consultations for all [42].

It is important to incorporate economic and rehabilitation programs for PAL to alleviate economic burden faced due to leprosy. Previous studies from Indonesia and India have shown interventions like microcredits and vocational training to be effective in providing financial independence and restoring social and economic status of PAL. Thereby, stigma is reduced and the overall quality of life of PAL enhanced [43,44].

Overall, this study revealed important aspects to be considered in Pakistan’s efforts to control and eliminate leprosy, reduce diagnostic delay and improve the well-being of those affected. Further research informed by the voices of PAL is needed to inform people-driven interventions on the path towards leprosy elimination in Pakistan and globally.

### Limitations

This study contains a few limitations that need to be considered. Although translators helped to facilitate the interviews, there may still have been language barriers. The use of translators may have influenced participants’ depth of responses, despite assured confidentiality. In addition, PAL may have been reluctant to share negative experiences related to their treatment experience at MALC due to the interviewer’s distant affiliation with the NGO. It is also important to note that the interpretation of presented findings may be influenced by biases arising from partially inductive coding and the adaption of the framework used to suit the context of the study. Furthermore, the study population includes only those physically and mentally able to participate and mostly those above the age of forty years.

## Conclusion

This study highlighted the lived experiences of those affected by leprosy, by exploring their care-seeking and treatment experience, well-being, stigma encounters and coping mechanisms. While Sindh accounts for the majority of cases in Pakistan, the overall prevalence of the disease remains low, contributing to diagnostic delay and lack of awareness. To overcome displayed barriers, interventions should be implemented on interpersonal, intrapersonal, community and institutional levels. In order to improve and honor the stories of PAL, it is therefore crucial to design people-driven interventions that empower and support PAL through storytelling, education, digital health tools and psychosocial counselling and adapted to their needs and beliefs.

## Supporting information

S1 Table Interview Guide. (DOCX)

S1 Checklist Inclusivity in global research questionnaire. (DOCX)

## References

[1] Leprosy Mission. Does leprosy still exist?. https://www.leprosymission.org/what-is-leprosy/does-leprosy-still-exist/. 2025. 2025 April 16.

[2] WHO. Leprosy. https://www.who.int/news-room/fact-sheets/detail/leprosy 2025. 2025 April 16.

[3] WHO. Leprosy (Hansen disease). https://www.who.int/health-topics/leprosy. 2025. Accessed 2025 March 27.

[4] CDC. Clinical Overview of Hansen’s Disease (Leprosy). https://www.cdc.gov/leprosy/hcp/clinical-overview/index.html. 2024. Accessed 2025 March 27.

[5] Fastenau A, Beresford MO, Willis M, Stuetzle SC, Schlumberger F, Duighuisen HNW. Understanding reasons for delay in diagnosis of leprosy in Pakistan: A qualitative study. PLoS Negl Trop Dis. 2025;19(1):e0012764. doi: 10.1371/journal.pntd.0012764 39774341 PMC11706370

[6] Santacroce L, Del Prete R, Charitos IA, Bottalico L. Mycobacterium leprae: A historical study on the origins of leprosy and its social stigma. Infez Med. 2021;29(4):623–32. doi: 10.53854/liim-2904-18 35146374 PMC8805473

[7] Saraswat N, Agarwal R, Chopra A. Assessment of factors responsible for dropout to multi drug therapy for leprosy. Indian J Lepr. 2019;91:225–32.

[8] National Health Mission. National Leprosy Eradication Programme. (NLEP):: National Health Mission, https://nhm.gov.in/index4.php?lang=1&level=0&linkid=281&lid=348 2025. accessed 8 April 2025.

[9] Government of Nepal. National Leprosy Strategy 2078-82 Nepali, https://edcd.gov.np/resource-detail/national-leprosy-strategy-2078-82-nepali 2023, Accessed 8 April 2025.

[10] Sebong PH, Ferdiana A, Tegu FAR, Harbianto D, Soviandhi R, Sinaga A, et al. Participatory development of Indonesia’s national action plan for zero leprosy: strategies and interventions. Front Public Health. 2025;13:1453470. doi: 10.3389/fpubh.2025.1453470 40276344 PMC12018332

[11] WHO. Leprosy, number of new cases globally. https://apps.who.int/neglected_diseases/ntddata/leprosy/leprosy.html. 2025. Accessed 2025 April 16.

[12] MALC – Marie Adelaide Leprosy Centre. https://malc.org.pk/. 2025. Accessed 2025 April 16.

[13] Aid to Leprosy. About Us. https://alp.com.pk/about/. 2023. Accessed 2025 April 16.

[14] Marie Adelaide Leprosy Centre (MALC). National Leprosy Data - Pakistan 2001–23.

[15] Mull JD, Wood CS, Gans LP, Mull DS. Culture and “compliance” among leprosy patients in Pakistan. Soc Sci Med. 1989;29(7):799–811. doi: 10.1016/0277-9536(89)90079-8 2799423

[16] Han E, Shiraz F, Haldane V, Koh JJK, Quek RYC, Ozdemir S, et al. Biopsychosocial experiences and coping strategies of elderly ESRD patients: a qualitative study to inform the development of more holistic and person-centred health services in Singapore. BMC Public Health. 2019;19(1):1107. doi: 10.1186/s12889-019-7433-6 31412824 PMC6694659

[17] Mukerji R, Turan JM. Exploring Manifestations of TB-Related Stigma Experienced by Women in Kolkata, India. Ann Glob Health. 2018;84(4):727–35. doi: 10.9204/aogh.2383 30779523 PMC6748300

[18] WHO. Towards zero leprosy. Global leprosy (Hansen’s Disease) strategy 2021–2030. 2021. https://www.who.int/publications/i/item/9789290228509

[19] WHO. SDG Target 3.3 Communicable diseases, 2025 https://www.who.int/data/gho/data/themes/topics/sdg-target-3_3-communicable-diseases 27 March 2025.

[20] Government of Pakistan. About Sindh Province. https://www.pppunitsindh.gov.pk/aboutsindh.php. 2025. 2025 March 27.

[21] Palinkas LA, Horwitz SM, Green CA, Wisdom JP, Duan N, Hoagwood K. Purposeful Sampling for Qualitative Data Collection and Analysis in Mixed Method Implementation Research. Adm Policy Ment Health. 2015;42(5):533–44. doi: 10.1007/s10488-013-0528-y 24193818 PMC4012002

[22] Willig C, Rogers WS. The SAGE Handbook of Qualitative Research in Psychology. London: SAGE. 2017.

[23] Varkevisser CM, Lever P, Alubo O, Burathoki K, Idawani C, Moreira TMA, et al. Gender and leprosy: case studies in Indonesia, Nigeria, Nepal and Brazil. Lepr Rev. 2009;80(1):65–76. doi: 10.47276/lr.80.1.65 19472853

[24] Peters R, Lusli M, Zweekhorst M. Learning from a leprosy project in Indonesia: making mindsets explicit for stigma reduction. Dev Pract. 2015;25:1105–19.

[25] Abdul Rahman N, Rajaratnam V, Burchell GL, Peters RMH, Zweekhorst MBM. Experiences of living with leprosy: A systematic review and qualitative evidence synthesis. PLoS Negl Trop Dis. 2022;16(10):e0010761. doi: 10.1371/journal.pntd.0010761 36197928 PMC9576094

[26] Fastenau A. Neglect of mental health issues and lack of integration of psychosocial interventions in Zero Leprosy Roadmaps: A critical oversight. PLOS Ment Health. 2024;1(4):e0000140. doi: 10.1371/journal.pmen.0000140 PMC12798547 41661814

[27] Bonkass A-K, Fastenau A, Stuetzle S, Boeckmann M, Nadiruzzaman M. Psychosocial interventions for persons affected by Leprosy: A systematic review. PLOS Ment Health. 2024;1(3):e0000091. doi: 10.1371/journal.pmen.0000091 PMC12798225 41661711

[28] Alvi MH, Ashraf T, Naz F, Sardar A, Ullah A, Patel A, et al. Burden of mental disorders by gender in Pakistan: analysis of Global Burden of Disease Study data for 1990-2019. BJPsych Bull. 2023;48(6):1–8. doi: 10.1192/bjb.2023.76 37772484 PMC11669452

[29] Khan SI, Irfan M. Stigmatization and Self-Perception regarding issues related to Mental Health: A qualitative survey from a lower and middle-income country. Pak J Med Sci. 2023;39(5):1411–5. doi: 10.12669/pjms.39.5.7487 37680841 PMC10480727

[30] Chatmon BN. Males and Mental Health Stigma. Am J Mens Health. 2020;14(4):1557988320949322. doi: 10.1177/1557988320949322 32812501 PMC7444121

[31] Obindo T, Eaton J, Tsaku P, Nwefoh E, Ode P, Baird T, et al. Integrated services for neglected tropical diseases and mental health: pilot study assessing acceptability, feasibility and attitudes in Benue State, Nigeria. Int Health. 2023;15(15 Suppl 3):iii37–46. doi: 10.1093/inthealth/ihad073 38118157 PMC10732684

[32] Willis M, Fastenau A, Penna S, Klabbers G. Interventions to reduce leprosy related stigma: A systematic review. PLOS Glob Public Health. 2024;4(8):e0003440. doi: 10.1371/journal.pgph.0003440 39172813 PMC11340997

[33] Nganda M, Luhaka P, Kukola J, Ding Y, Bulambo C, Kadima J, et al. Participatory development of a community mental wellbeing support package for people affected by skin neglected tropical diseases in the Kasai province, Democratic Republic of Congo. Int Health. 2024;16(16 Suppl 1):i30–41. doi: 10.1093/inthealth/ihae008 38547352 PMC10977949

[34] Nayak PK, Mackenzie CD, Agarwal A, van Wijk R, Mol MM, Eaton J, et al. A new guide for basic psychological support for persons affected by neglected tropical diseases: A peer support tool suitable for persons with a diagnosis of leprosy and lymphatic filariasis. PLoS Negl Trop Dis. 2025;19(1):e0011945. doi: 10.1371/journal.pntd.0011945 39787071 PMC11717307

[35] Susanto T, Dewi EI, Rahmawati I. The experiences of people affected by leprosy who participated in self-care groups in the community: A qualitative study in Indonesia. Lepr Rev. 2017;88:543–53.

[36] Lusli M, Peters R, van Brakel W, Zweekhorst M, Iancu S, Bunders J, et al. The Impact of a Rights-Based Counselling Intervention to Reduce Stigma in People Affected by Leprosy in Indonesia. PLoS Negl Trop Dis. 2016;10(12):e0005088. doi: 10.1371/journal.pntd.0005088 27959932 PMC5154499

[37] Joo JH, Bone L, Forte J, Kirley E, Lynch T, Aboumatar H. The benefits and challenges of established peer support programmes for patients, informal caregivers, and healthcare providers. Fam Pract. 2022;39(5):903–12. doi: 10.1093/fampra/cmac004 35104847 PMC9508871

[38] Hotopf I, Chowdhury S, Robert G. Community-based models for neglected tropical diseases affecting the skin: a scoping review. Front Trop Dis. 2025;6:1544842.

[39] Correia JC, Golay A, Lachat S, Singh SB, Manandhar V, Jha N, et al. “If you will counsel properly with love, they will listen”: A qualitative analysis of leprosy affected patients’ educational needs and caregiver perceptions in Nepal. PLoS One. 2019;14(2):e0210955. doi: 10.1371/journal.pone.0210955 30726259 PMC6364891

[40] Lima MCV, Barbosa FR, Santos DCMD, Nascimento RD do, D’Azevedo SSP. Practices for self-care in Hansen’s disease: face, hands and feet. Rev Gaucha Enferm. 2018;39:e20180045. doi: 10.1590/1983-1447.2018.20180045 30365764

[41] Sottie CA, Darkey J. Living with stigma: Voices from the Cured Lepers’ village in Ghana. Soc Work Health Care. 2019;58(2):151–65. doi: 10.1080/00981389.2018.1526842 30321131

[42] Barnowska EJ, Fastenau A, Penna S, Bonkass A-K, Stuetzle S, Janssen R. Diagnosing skin neglected tropical diseases with the aid of digital health tools: A scoping review. PLOS Digit Health. 2024;3(10):e0000629. doi: 10.1371/journal.pdig.0000629 39374195 PMC11458012

[43] Rao VP, Rao IR, Palande DD. Socio-economic rehabilitation programmes of LEPRA India--methodology, results and application of needs-based socio-economic evaluation. Lepr Rev. 2000;71(4):466–71. doi: 10.5935/0305-7518.20000048 11201901

[44] Dadun D, Van Brakel WH, Peters RMH, Lusli M, Zweekhorst MBM, Bunders JGF, et al. Impact of socio-economic development, contact and peer counselling on stigma against persons affected by leprosy in Cirebon, Indonesia –a randomised controlled trial. Lepr Rev. 2017;88(1):2–22. doi: 10.47276/lr.88.1.2 30188085

