# Supplementary material for: “The general public will never truly know what it feels like”: Exploring the Lived Experiences of Persons Affected by Leprosy in Sindh, Pakistan – A qualitative study
Source: PLOS Ment Health. 2025 Nov 5;2(11):e0000453. doi: 10.1371/journal.pmen.0000453 (PMC12798628; doi:10.1371/journal.pmen.0000453)
Supplement: S1 Table — (DOCX) [file pmen.0000453.s001.docx]

**SI 1** Table 2 Interview Guide

Introduction

1. Thank you for taking your time to participate in this interview/ study.
2. Short Introduction of my person/translator
3. Short explanation of the research project/ aims
4. The interview will approximately take 30-90 minutes/ longer/shorter if necessary.
5. Confirmation of consent form and data protection form

Have you read, understood and signed the consent and information form? Are there any remaining questions concerning the forms that you would like to address or is everything clear?

1. Ask for approval to record the interview

As you have already read in the consent form, I would like to record our interview/ conversation for data analysis purposes later on. All recorded information will be later transcribed, pseudonymized and the recordings will then be deleted. I want to double check whether you agree with this procedure or if you would be more comfortable if I only took notes?

1. Renewed assurance of pseudonymization

Before we start, I would like to reassure you that everything you are sharing with me today will remain anonymous and won’t be able to be traced back to you.

*I would like to talk about your experiences with leprosy today. I understand that these are sensitive topics, and some of these questions may bring to mind painful/difficult experiences. You are welcome to share only what you feel comfortable with and can stop or take breaks at any time. If you have difficulties understanding some of the questions, please don’t hesitate to ask any time. I will take some notes for myself so I can better remember what we discussed.*

| Theme | Question | Follow-up/Examples | comments |
| --- | --- | --- | --- |
| **1. General Information** | How old are you? |  |  |
|  | What is your job/education? | If no job, why is that? Because of leprosy? |  |
|  | Are you married? |  |  |
|  | Do you have children? |  |  |
|  | Where are you from? Do you live in Karachi? |  | Relevant, as there are oftentimes differences between communities |
| **2. History of Diagnosis & Internalized Stigma** | How long have you been living with the consequences of leprosy? |  |  |
|  | Can you tell me a little bit about your life before you became sick? | Have “these things” changed since then? |  |
|  | When did you notice changes in your body (due to leprosy)? | What happened after you noticed these changes? |  |
|  |  | How did you feel? |  |
|  |  | Did you tell someone? If yes, who? If no, why? |  |
|  | When and why did you decide to seek care? | Where did you decide to seek care/help first? |  |
|  |  | Have you looked for help/care elsewhere? E.g., a healer or similar? If yes/no, why? |  |
|  | What was your treatment like? | How did you feel when you heard the news about your illness? |  |
|  |  | How did the doctor/caretaker make you feel about your illness? |  |
|  | Do you know what medicines the doctors gave you for your illness? | How does/did that medicine make you feel? |  |
| **3. General Knowledge & Internalized Stigma** | Had you ever heard about the illness leprosy before your symptoms? | If yes, where did you hear about it? |  |
|  | Why do you think you got sick with leprosy? | Why do you think that/Where did you learn that? |  |
|  | Why do you think some people get leprosy and others don’t? | Why do you think that/Where did you learn that? |  |
|  | Can you tell me about what leprosy feels like in your body and mind? | What is it like compared to other times that you were sick in your life? |  |
|  | Do you think your illness is curable? | If no, why? If yes, did you think that before you started treatment? |  |
| **4. Experienced Stigma & Internalized Stigma** | Can you tell me about your days currently, from when you wake up to when you go to sleep, how does your day go? | Have you had to make changes, e.g., your work? |  |
|  | Can you tell me something about your community, your family, friends, work, people at the mosque, how do you feel about telling them about your illness? How do you feel about them seeing you sick? | Have you told anyone, if yes, who and when? If no, why not? | Adapted from EMIC-CC/SARI |
|  |  | How did they react when you told them? How did that make you feel? |  |
|  |  | Do they treat you differently? If yes, Can you understand that they treat you differently? If yes, why? |  |
|  |  | Would you like to have more support from them? |  |
|  | Do you know anyone else that has leprosy? | If yes, how do you know them? If no, would you like to know others and why? | Adapted from EMIC-CC/SARI |
|  | Would you describe your life as good and fulfilling these days or would you like to change anything in your day-to-day life with leprosy? | If yes, why? |  |
| **5. Anticipated Stigma & Local Beliefs** | Are there any stories/legends/stereotypes about persons affected by leprosy or leprosy in your community? | What kind of stories? Can you tell me one of these stories? | PST-toolkit |
|  |  | Do you agree with that or not, and why? |  |
|  | Do people have the same chance of finding a job if affected by leprosy? | Why not? Does it matter whether you were treated/ untreated? | Adapted from EMIC-CC/SARI |
|  | Does having/had leprosy affect people’s access to public places/facilities? | Why? Does it matter whether you were treated/ untreated? | Adapted from EMIC-CC/SARI |
|  | How do people feel around someone affected by leprosy? | Why? Does it matter whether you were treated/ untreated? | Adapted from EMIC-CC/SARI |
|  | In what way would having leprosy influence someone’s chance to get married? | Why? Does it matter whether you were treated/ untreated? | Adapted from EMIC-CC/SARI |
|  | Does having/had leprosy influence people’s decision to visit one’s home? | Why? Does it matter whether you were treated/ untreated? | Adapted from EMIC-CC/SARI |
|  | In what way do religious beliefs/and or teaching influence people’s perception of leprosy? | How? |  |
|  | What is stigma to you? | Please elaborate |  |
|  | What does leprosy embody to you? | Please elaborate |  |
| **6. Recommendations** | Is there anything you would like to say to your community/ colleagues/ family/HCWs about leprosy? | Do you feel you are not able to say it openly to them? If yes, why? | *Reminder: anonymous* |
|  | Would you (have) like(d) to change something in your treatment/diagnosis? | If yes, why? If no, what was good about it? | *Reminder: anonymous* |
|  | Is there anything that could (have) help(ed) you in your day-to-day life with leprosy? | If yes, what? |  |
| **6. Review/Closing** | Brief summary of the interview |  |  |
|  | Is there anything else you would like to add? |  |  |
|  | Do you have any remaining questions? |  |  |
|  | Thank you for your time and participation. The information that you have shared will greatly contribute to the research project. | Renewed assurance of pseudonymization |  |

Disclaimer

- General information about leprosy (transmission, cause, treatment etc.)
- Having (had) leprosy is in no shape or form your fault.
- Reminder for psycho-social support by MALC
- Reminder for treatment options at MALC

**References**

Boyd, J.E., Adler, E.P., Otilingam, P.G., Peters, T. (2014) Internalized Stigma of Mental Illness (ISMI) scale: a multinational review. *Compr Psychiatry*, 55(1):221-31.

Infolep. (2024). Perception Study Toolkit (PST). <https://www.leprosy-information.org/toolkits/perception-study-toolkit-pst-0>.

InfoNTD. (2024). SARI Stigma- Scale. <https://www.infontd.org/toolkits/ntd-morbidity-and-disability-toolkit/sari-stigma-scale>.

InfoNTD. (2024). EMIC-CS Emic Community Stigma. <https://www.infontd.org/toolkits/ntd-morbidity-and-disability-toolkit/emic-cs-emic-community-stigma>.

InfoNTD. (2024). SDS Social Distance Scale. <https://www.infontd.org/toolkits/sds-social-distance-scale>.
